# Apremilast Regulates the Teff/Treg Balance to Ameliorate Uveitis *via* PI3K/AKT/FoxO1 Signaling Pathway

**DOI:** 10.3389/fimmu.2020.581673

**Published:** 2020-11-17

**Authors:** Yuxi Chen, Zhuang Li, He Li, Wenru Su, Yanyan Xie, Yuan Pan, Xiaoqing Chen, Dan Liang

**Affiliations:** State Key Laboratory of Ophthalmology, Zhongshan Ophthalmic Center, Sun Yat-sen University, Guangzhou, China

**Keywords:** apremilast, uveitis, PI3K/AKT/FoxO1 signal pathway, Teff/Treg, PDE4, experimental autoimmune uveitis

## Abstract

Autoimmune uveitis (AU), being one of the sight-threatening ocular inflammatory disorders, has been widely regarded by ophthalmologists and immunologists as a great challenge. Apremilast, a phosphodiesterase-4 inhibitor (PDE4i), which was approved by the U.S. Food and Drug Administration (FDA) for the treatment of active psoriatic arthritis in 2014, has been attracting researchers, who are exploring its efficiency and mechanism on uveitis. In this study, we used an experimental autoimmune uveitis (EAU), a representative model for human AU, to investigate the effect of apremilast on regulating anti-inflammatory mediators. Our study demonstrated that apremilast treatment resulted in a decrease in vascular leakage, macular edema, and inflammatory cell infiltration in the retina, corresponding to decreased clinical and pathological scores. Specifically, apremilast decreased the proportion and population of Th17 cells and increased the proportion and population of T regulatory (Treg) cells. Mechanistically, apremilast may regulate Th17 and Treg cells by inhibiting the phosphorylation of the phosphoinositide 3-kinase (PI3K)/protein kinase B(AKT)/Forkhead box O1 (FoxO1) signaling pathway. These findings suggested that apremilast alleviated EAU by regulating Th17 and Treg through the PI3K/AKT/FoxO1 pathway.

## Introduction

Autoimmune uveitis (AU) has been widely considered as one of the ocular inflammatory disorders that cause significant damage to vision (1). Glucocorticoids, immune-suppressants, or biological agents, which are used for uveitis, are still faced with huge challenges, including restricted efficiency and long-term adverse events (2, 3). Therefore, it is necessary to search for new medications that could treat this refractory sight-threatening disease.

Apremilast, a small molecule-inhibiting phosphodiesterase-4(PDE4), has been confirmed to be able to regulate downstream inflammatory mediators by preventing cyclic adenosine monophosphate (cAMP) degradation into adenosine monophosphate (AMP) (4). cAMP plays an essential role as a second messenger in diverse intracellular pathways, involving many metabolic reactions and inflammatory activities (5). Convincingly, apremilast is supposed to act as a therapeutic agent for various autoimmune disorders by increasing intracellular cAMP (6). The effect of apremilast on several chronic inflammatory disorders, such as psoriasis (7, 8), dermatitis (9), and rheumatoid arthritis (10, 11, 12) has been studied and verified in animal models. Inspiringly, apremilast was approved by the U.S. Food and Drug Administration (FDA) in 2014 to treat adult active psoriatic arthritis (13, 14). To search for new drugs for AU, we determined to explore the effects of apremilast on uveitis.

PI3Ks can be classified into three subtypes and are involved in various biochemical reactions, including cell growth, survival, differentiation, material transport, and metabolism (15). AKT, belonging to the AGC family of serine/threonine protein kinase, acts as the main downstream molecule of PI3K signaling (16). The PI3K/AKT pathway plays a core role in the process and release of pro-inflammatory factors (17, 18). Few studies confirmed the relationship between PED4 inhibitors (including apremilast) and PI3K/AKT. Peter G. Smith et al. confirmed PDE4B is one risk-related factor in an independent series of primary DLBCLs, and cAMP-mediated apoptosis in DLBCL is related to inhibition of the PI3K/AKT pathway (19). A single-center, exploratory phase Ib open-label, nonrandomized study was further performed, and it showed that the PDE4 inhibitor roflumilast is a safe therapy for treatment of B-cell malignancies by suppressing the activity of the oncogenic PI3K/AKT kinases (20). Torbafylline, another PDE inhibitor, was illustrated to attenuate burn-induced rat skeletal muscle proteolysis through the PDE4/cAMP/EPAC/PI3K/Akt pathway. Phosphodiesterase (PDE) inhibitor torbafylline (HWA 448) attenuates burn-induced rat skeletal muscle proteolysis through the PDE4/cAMP/EPAC/PI3K/Akt pathway (21). It is interesting to explore whether, in experimental AU (EAU), apremilast could influence the PI3K/AKT pathway. In addition, transcription factor forkhead-box O1 (FoxO1), among the FoxO family in mammals, is modulated mainly by the PI3K/AKT signal via phosphorylation (22), and FoxO1 has been reported to regulate several downstream gene targets including pro-inflammatory molecules, adhesion molecules, B-cell regulators, and T-regulatory modulators (23). It has been recently shown to increase Foxp3 expression of CD4+ T-cells and strengthen the population and capability of Treg cells (24). The imbalance between regulatory CD4+ T (Treg) and effector CD4+ T-cells (Teffs, such as Th17 and Th1) has been widely accepted to be the core mechanism of autoimmune diseases including AU (2). The key to controlling the inflammation of AU is to rebuild the balance by strengthening the population and capability of Treg cells. We sought to understand whether apremilast could modulate Treg/Teff cells to ameliorate EAU via the PI3K/AKT/FoxO1 pathway.

## Materials and Methods

### Animals

Adult female C57BL/6J mice, age 6–8 weeks and weighing 20–25 g, were purchased from the Guangzhou Animal Experiment Center. Each animal used for the study was bred in a specific pathogen-free condition with a 12-h light–dark cycle as well as steady temperature and humidity. During the whole breeding and experimental period, clean food and water were provided to the mice. Mouse experiments were strictly conducted according to the guidelines of the Institutional Animal Care Committee affiliated to Zhongshan Ophthalmic Center, Sun Yat-sen University.

### Establishment of EAU Model

Complete Freund’s adjuvant (CFA, BD Difco, San Jose, CA, USA) containing 5 mg Mycobacterium tuberculosis H37Ra (BD Difco, San Jose, CA, USA) was mixed with 200 μg hIRBP1-20 (GPTHLFQPSLVLDMAKVLLD, GL Biochem, Shanghai, China) in a 1:1 volume ratio (v/v). The emulsion was injected into the back spot of each mouse, near the tail and two flanks subcutaneously. In addition, 250 ng Bordetella pertussis toxin (PTX, List Biological Laboratories, Campbell, California, USA) dissolved in phosphate-buffered saline (PBS, Gibco, Grand Island, New York, USA) was intraperitoneally injected to the immunized mice on days 0 and 2 after immunization (25–27).

### Treatment of EAU Model

Apremilast, at a purity of more than 95% purchased from Selleck Chemicals (Houston, TX, USA), was dissolved into dimethyl sulfoxide (DMSO, Sigma, 0.1%) for storage at -80°C. It was further dissolved by PBS containing carboxymethyl cellulose (CMC, Sigma, 0.5%) and Tween 80 (Sigma, 0.25%) for in vivo and in vitro experiments (11).

All the immunized mice were administrated with vehicle (0.1% DMSO, 0.5% CMC, 0.25% Tween 80) or apremilast at different dosages (5, 15, or 25 mg/kg) by oral gavage from days 7 to 21 after immunization (11).

### Clinical and Histopathologic Evaluation of Retina Inflammation

On the 21st day after immunization, fundus photos were taken to observe the retinal hemorrhage, vascular leakage, macular edema, retinal folds, retinal detachment, etc. Clinical scores were graded from 0 to 4 according to the fundus images (Phoenix Co., Campbell, California, USA) (27, 28).

On the 21st day after immunization, the eyeballs gathered from the experimental mice were fixed in 4% PFA at room temperature for 48 h and paraffin embedded. The fixed eyeballs were cut into 5-μm-thick slides at the margin of the optic nerve for hematoxylin and eosin (H&E) staining. Histopathologic manifestations, including inflammatory cell infiltration and the retinal folds of each retina level, were photographed and evaluated with a score from 0 to 4 (27, 28).

### Isolation of Retinal Infiltrated Cells

To isolate retinal cells, the eyeballs were removed from the orbital cavity of the mice and were temporarily stored in an RPMI-1640 culture medium (Gibco, USA) with 10% fetal bovine serum (FBS, Gibco) on ice. Retinas were dissected from the eyes under the microscope and were then polished and cut into small pieces. To deeply digest the retinas into single cell suspensions, they were incubated at 37° for 60 min in an RPMI-1640 culture medium containing collagenase D (Roche, Basel, Switzerland) and 10% FBS. Finally, after washing twice with PBS, the retinal infiltrated cells were obtained for flow cytometry analysis (29).

### Isolation and Treatment of Draining Lymph Nodes Cells

To isolate the cells of draining lymph nodes (DLNs), inguinal, axillary, and cervical lymph nodes were harvested from the mice on day 21 after immunization and ground. Cells were filtered through a cell strainer to make single-cell suspensions. All the cells were plated in a 96-well plate (approximately 5*10^5^ cell/well) and incubated with IRBP1-20 (20 μg/ml) at 37°C for 72 h in a humidified incubator with 5% CO_2_. After treatment with different doses of apremilast (0, 50, 100, 200, 400, and 800 nM), the cells were analyzed by flow cytometry.

### Flow Cytometry

To test the CD4+ lymphocytes, LIVE/DEAD (Thermo Fisher Scientific, Waltham, MA, USA) was first labeled to distinguish the living cells. Surface marker antimouse CD4 and CD45 were labeled to all the cultured cells. After further fixation and permeabilization were performed for intracellular staining, intracellcular inflammatory cytokines were labeled and evaluated using an LSRFortessa (BD Biosciences). The following antimouse antibodies used for the cytometry analysis were purchased from BioLegend (San Diego, CA, USA) or Abcam (Cambridge, MA, USA) including antimouse CD4 Percp-cy5.5 (Biolegend), antimouse CD45 BV510 (Biolegend), antimouse IL17A APC (Biolegend), antimouse IFN-γ PE (Biolegend), antimouse TNFα BV421 (Biolegend), antimouse Foxp3 FITC (Biolegend), antimouse pPI3K PE (Biolegend), antimouse pAKT APC (Biolegend), antimouse FoxO1 (Abcam), and antimouse pFoxO1 (Abcam). SC79 (Selleck Chemicals, Houston, TX), as an AKT activator, was also used to stimulate the cells isolated from the DLNs and spleens of the EAU group to further determine whether the PI3K/AKT/FoxO1 pathway was involved in the mechanism of apremilast treatment on EAU. All the figures and data were collected and analyzed with FlowJo software (Tree Star, Ashland, OR).

### EAU Induction by the Adoptive Transfer Therapy

To further investigate the effect of apremilast on pathological IRBP-specific CD4+ T-lymphocytes, we designed a T-lymphocyte adoptive transfer experiment. Healthy C57BL/6J mice were chosen as recipients and were divided into three groups: healthy control group (CT), apre- adoptive transfer group (apre- AT), and apre+ adoptive transfer group (apre+ AT). Cells were separated from the inguinal, axillary, and cervical LNs of EAU on the 14th day after immunization. After being co-cultured with/without apremilast and stimulated with IRBP1-20 (20 μg/ml) for 72 h in a 96-well plate, CD4+ T-cells were sorted, purified, and collected. Recipient mice of the apre+ AT or apre-AT groups, respectively, received CD4+ T-cells co-cultured with or without apremilast through tail vein injection (2×10^7^ living cells/mouse). Mice of the CT received an equal volume of PBS. Different groups of mice were raised under the same condition (as in 1.1 animals).

### Statistical Analysis

Student’s *t* test and Mann-Whitney test, as well as one-way ANOVA were all performed for statistical analysis using the Graph Pad Prism software (Version 8.0, La Jolla, CA, USA). All the data were presented as mean±SD and *P*lt;0.05 was considered to be statistically significant.

## Results

### Apremilast Alleviated EAU Clinically and Histologically

**Figure 1:**
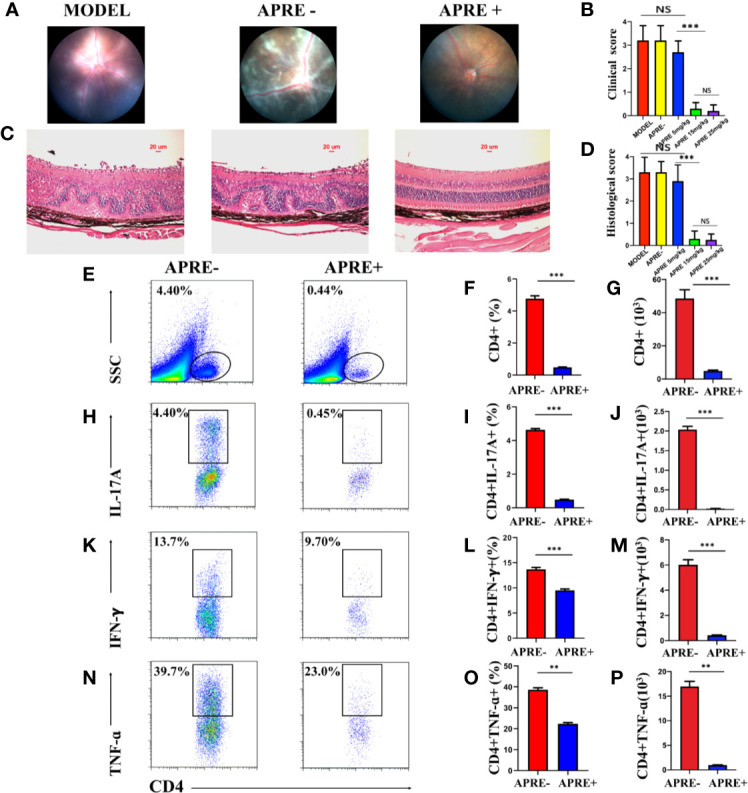
Apremilast ameliorated the inflammation of EAU. **(A)** The typical fundus images of the different administration on the 21st day after immunization. **(B)** Clinical scores chart of the five groups. **(C)** The representative histological images of H&E stain on the 21st day after immunization. **(D)** Histological scores of five groups. **(E–P)** The frequency and number of CD4+ T-cells, IL-17A-, and IFN-γ-producing cells that infiltrated the retina were measured by flow cytometry on day 21 after immunization. Apremilast blocked the CD4+ T-cells from entering the retina and reduced the frequency and number the of IL-17A-and IFN-γ-producing cells. Data expressed the mean ± SD, **P < 0.01, ***P < 0.001. *N* = 6.

To determine the therapeutic effect of apremilast on EAU, different doses of apremilast (5, 15, or 25 mg/kg) or vehicle were orally administered to the mice from the 7th day after immunization. On day 21, fundus photos were taken to evaluate the clinical scores of their fundus. Eyeballs were taken to make sections for pathological scores. Compared with the EAU mice of the vehicle (apre- group), apremilast treatment decreased the ocular inflammation features of the chorioretinal lesions, vascular leakage, and vasculitis dose-dependently. Representative fundus images are shown in **Figure 1A**. We found that apremilast at 5 mg/kg had little effect to alleviate EAU. However, apremilast at 15 and 25 mg/kg showed obvious better efficacy on EAU although no significant different effect between the two groups (**Figure 1B**). Apremilast treatment also decreased the retina folds and inflammatory cell infiltrations, which were summarized and calculated as pathological scores (**Figure 1C**). The pathological scores revealed consistent dosage effects of apremilast with the clinical scores (**Figure 1D**). Based on drug safety, we selected the dose of 15 mg/kg for the subsequent experiments. These results demonstrated that apremilast (15 mg/kg) reduced both the clinical and histological scores and then ameliorated the EAU manifestations strikingly and safely.

### Apremilast Prevented Teff Cells From Infiltrating Into Retina

It is widely well known that the infiltration of Teff cells, especially Th17 and Th1, into the retina is crucial in the pathologic retinal damage of EAU. To explore whether apremilast could prevent Teff cells from getting into the eyes, we isolated retinal infiltrated cells for further flow cytometry analysis. The population and proportion of CD4+ T-cells as well as inflammatory mediators, including IL17A, IFNγ, and TNFα in the retina were compared between the EAU mice of the apre+ and apre- groups. We found that CD4+ T-cells in the retina of the apre- group EAU mice appeared to be much more abundant than those in the retina of the apre+ group EAU mice (**Figures 1E–G**). Furthermore, Th17, Th1, and TNFα-producing cells isolated from the retina of the apre+ group EAU mice were fewer than those in the retina of the apre- group EAU mice (**Figures 1K–P**). Apremilast significantly decreased the migration of CD4+ T-cells, especially Th17, and IFNγ-producing cells into the retina. These results indicate that apremilast could prevent CD4+ T-cells, especially Teff cells, from infiltrating the retina and then alleviate uveitis.

### Apremilast Restored Teff/Treg Balance of EAU *in Vivo* and *in Vitro*

**Figure 2:**
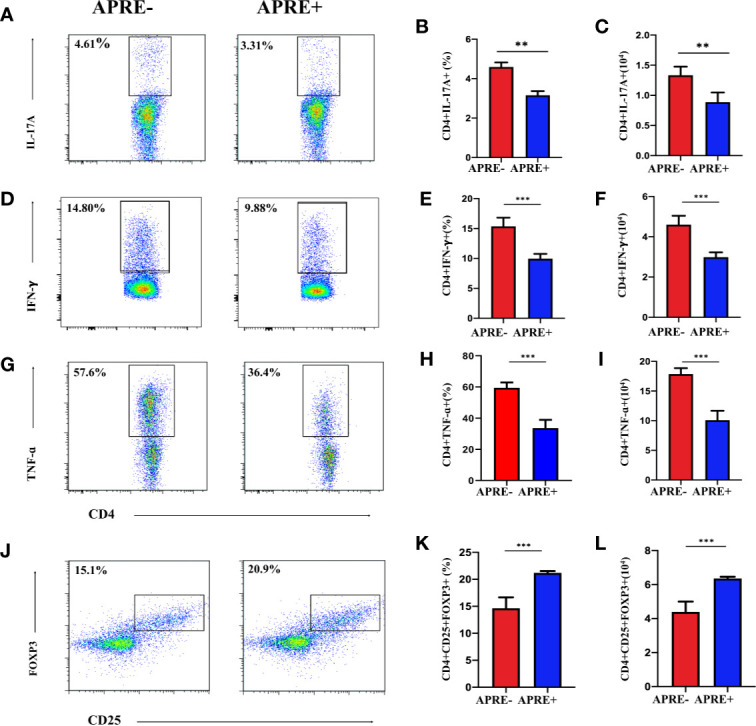
Apremilast inhibited Teff and strengthened Treg in the DLNs. CD4+ T-lymphocyte subtype on day 21 after immunization were compared between the apremilast treatment (15 mg/kg) and vehicle groups by flow cytometry quantitation. **(A–I)** The frequency and number of IL17A-, IFNγ-, and TNFα-producing cells. **(J–L)** The proportion and population of CD4+CD25+Foxp3+ T-cells. Data expressed as mean±SD, **P < 0.01, ***P < 0.001, *N* = 6.

Imbalance of Treg and Teff cells is regarded as one of the dominant mechanisms of uveitis. To evaluate whether apremilast could regulate Treg and Teff in vivo, we isolated T-cells from the DLNs of EAU mice from the apre+ and apre- groups and analyzed them by flow cytometry. We found that the frequency and number of IL17A-, IFNγ-, and TNFα-producing cells of the apre+ group were significantly lower than those of the apre- group, suggesting that apremilast suppressed Th17 and Th1 (**Figures 2A–I**). Meanwhile, higher frequency and number of Tregs (CD4+CD25+Foxp3+) were measured in the apre+ group (**Figures 2J–L**). These results show that apremilast can increase the population and proportion of Tregs and decrease the number and frequency of Teffs in EAU.

**Figure 3:**
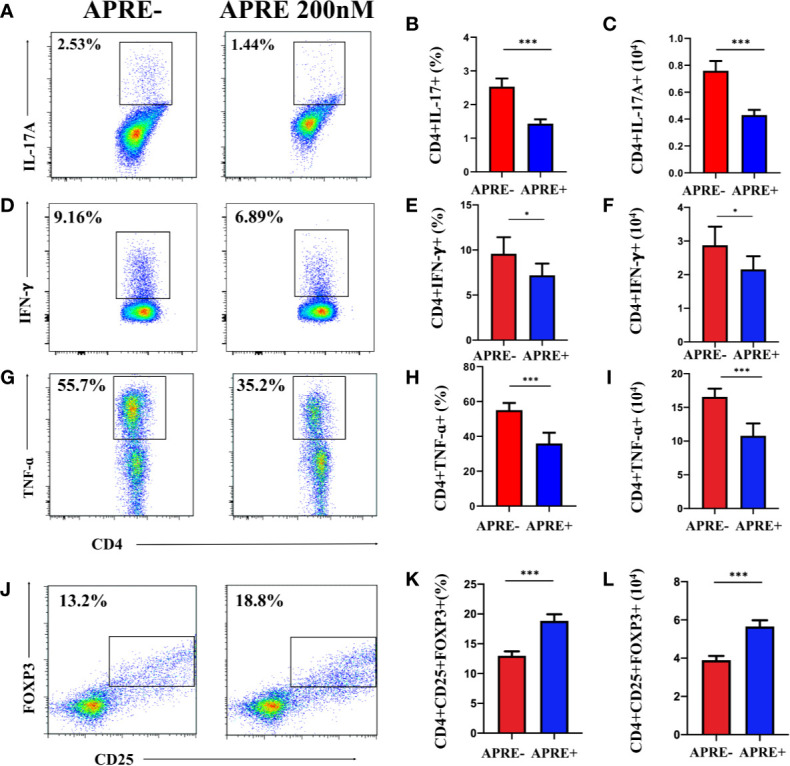
Apremilast modulated naïve CD4+ T-cells *in vitro*. Cells isolated from the DLNs stimulated with IRBP_1-20_ for 3 days with or without apremilast were analyzed by flow cytometry. **(A–I)** Apremilast suppressed the frequency and number of IL17A-, IFNγ-, and TNFα-producing cells. **(J–L)** Apremilast upregulated the proportion and population of CD4+CD25+Foxp3+ T-cells. Data expressed as mean±SD, **P < 0.01, ***P < 0.001, *N* = 6.

To further assess the ability of apremilast to regulate Treg and Teff in vitro, lymphocytes separated from the DLNs of EAU were cultured with or without apremilast. A gradient concentration of apremilast was applied in the DLNs, and 200 nM apremilast showed the greatest effects. As shown in **Figure 3**, apremilast decreased the proportion and population of Th17 and Th1 (**Figures 3A–I**) and increased Treg cells (**Figures 3J–L**). Overall, apremilast could regulate CD4+ T cells in vitro.

### Apremilast Ameliorated EAU by Modulating IRBP-Specific CD4+ T-Lymphocytes

**Figure 4:**
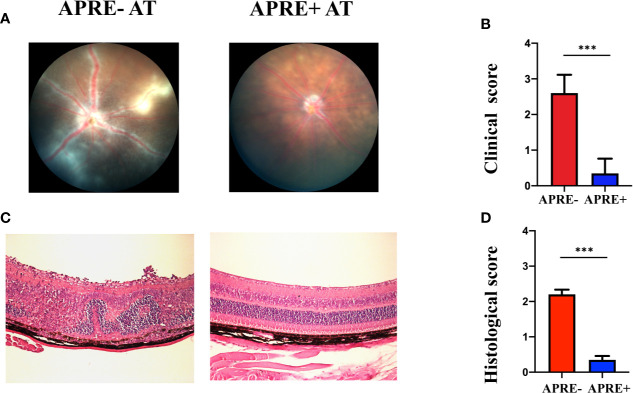
Apremilast prevented IRBP_1-20_ induced T-cells from entering the EAU. **(A, B)** On the 21st day after injection of the cultured IRBP_1-20_-specific T-cells treated with or without apremilast, typical fundus photographs were taken, and the two groups’ clinical scores were calculated. **(C, D)** The histological images and scores. Data expressed as mean±SD, ***P < 0.001, *N* = 6.

We conducted the adoptive transfer experiment to confirm whether apremilast could ameliorate EAU by regulating CD4+ T-cells. Mice of apre- AT, apre+ AT, and CT were injected with CD4+ T-cells co-cultured with or without apremilast or PBS through the tail vein. During the feeding period, fundus photos were taken to assess the clinical scores every 7 days, and eyeballs were taken to make sections for the pathological scores. Representative fundus photos of apre- AT and apre+ AT on day 21 are exhibited in **Figure 4A** (photos of the CT aere not shown). Mice of the apre+ AT group showed fewer ocular inflammation features, including chorioretinal lesions, vascular leakage, and vasculitis, than the mice of the apre- AT group (**Figure 4A**). There were fewer retina folds and inflammatory cell infiltrations in apre+ AT than those in apre- AT, which was identical results to the fundus images (**Figure 4C**). To sum up, apremilast treatment significantly decreased the inflammation of EAU induced by the adoptive transfer experiment clinically and pathologically (**Figures 4B, D**). This adoptive transfer experiment declared that apremilast ameliorated EAU by modulating IRBP-specific CD4+ T-lymphocytes.

### Apremilast Attenuated EAU via the PI3K/AKT/FoxO1 Signaling Pathway

**Figure 5:**
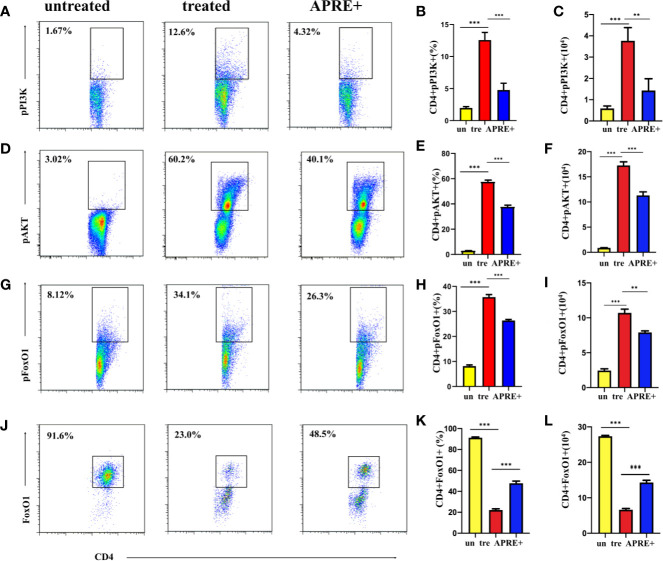
Apremilast attenuated the EAU via the PI3K/AKT/FoxO1 signaling pathway. **(A–I)** CD4+ T-lymphocytes from the DLNs of the EAU group induced by IRBP_1-20_ for 3 days with or without apremilast treatment were analyzed; the proportion and population of pPI3K+, pAKT+, and pFoxO1+. **(J–L)** The frequency and number of FoxO1+ CD4+ T-cells with or without apremilast treatment. Data expressed as mean±SD, **P < 0.01, ***P < 0.001, *N* = 6.

The PI3K/AKT pathway is reported to be important for its signal regulation function in several autoimmune diseases. PI3K/AKT are often dysregulated in autoimmune diseases, including AU. To investigate whether PI3K/AKT/FoxO1 is implicated in the therapeutic effect of apremilast on EAU, we evaluated the levels of pPI3K, pAKT, and pFoxO1 of the CD4+ T-lymphocytes of EAU with apremilast (200 nM). Meanwhile, we checked the level of FoxO1. We found that apremilast upregulated PI3K/AKT/FoxO1 phosphorylation of the CD4+ T-lymphocytes isolated from the EAU and suppressed the phosphorylation of the intracellular protein significantly (**Figures 5A–I**). In addition, FoxO1 was consistently upregulated by apremilast (**Figures 5J–L**).

**Figure 6:**
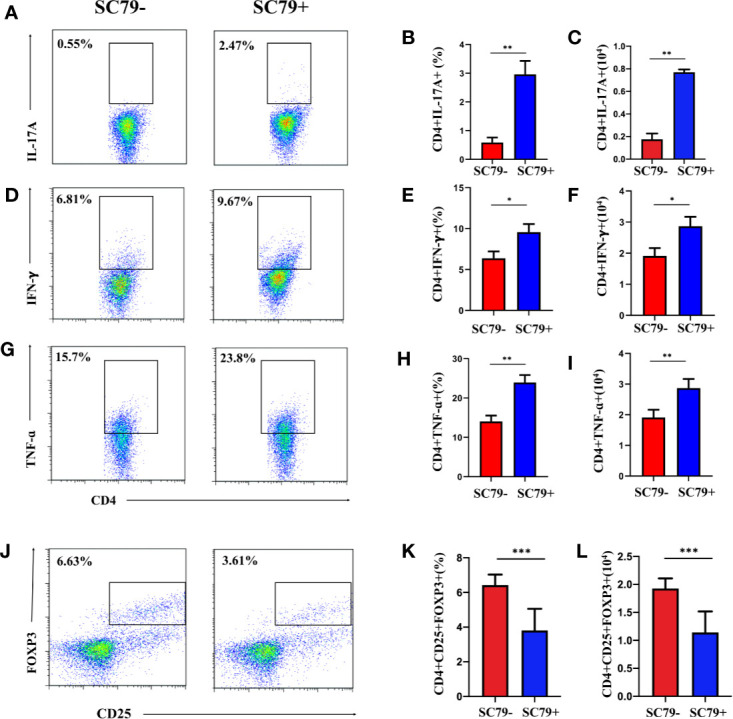
Apremilast failed to rebuild the imbalance between Treg/Teff of EAU co-stimulated with SC79. Cells from the DLNs of the EAU group were cultured in IRBP_1-20_ and apremilast with or without SC79 (16 mg/ml). **(A–I)** The proportion and population of IL17A-, IFNγ-, and TNFα-producing CD4+ T-cells in the two groups. **(J–L)** The proportion and population of CD4+CD25+Foxp3+ T-cells of the two groups. Data expressed as mean±SD, *P < 0.05, **P < 0.01, ***P < 0.001, *N* = 6.

SC79 is a specific AKT activator that can upregulate the phosphorylation of intracellular AKT and is often used to promote the phosphorylation of the PI3K/AKT pathway. We used SC79 to treat CD4+ T-cells from the DLNs of EAU with apremilast. We wanted to determine whether SC79 could reverse the effect of apremilast on downregulating inflammatory mediators. This experiment would also help us to confirm that the PI3K/AKT/FoxO1 pathway is involved in the apremilast’s therapeutic effect on EAU. Flow cytometry showed that SC79 promoted the expression of inflammatory mediators, including IL17A, IFNγ, and TNFα (**Figures 6A–I**), while it inhibited Treg (**Figures 6J–L**). These results indicate that the differentiation and function of CD4+ T-cells might be regulated by the phosphorylation of intracellular AKT. It is reasonable to presume that apremilast alleviated EAU by reducing the phosphorylation of the PI3K/AKT/FoxO1 pathway.

## Discussion

For the first time, this study shows that apremilast could treat AU effectively in an animal model. First, we demonstrate that apremilast could prevent CD4+ T-cells, especially Teff cells, from infiltrating the eyes of EAU mice. Next, we demonstrated that apremilast significantly suppressed Th17 and Th1 cells and enhanced Treg in the DLNs of the EAU group in vivo and in vitro, meaning that it could harmonize the balance between Teff/Treg induced by the autoimmune inflammation. We also demonstrate that phosphorylation and activation of the PI3K/AKT/FoxO1 pathway could act as a new pathogenic mechanism involved in EAU. Finally, we find that apremilast could reduce the phosphorylation of PI3K/AKT/FoxO1 to alleviate EAU.

In the past few years, the efficacy of PDE4 inhibitors on autoimmune diseases has been reported. R. Caspi et al. found that rolipram, the first selective PDE4 inhibitor, had a protective effect in an EAU model (30), and Zai-Long Chi et al. found that rolipram could inhibit the pathogenesis of LPS-induced uveitis (31). Roflumilast, a second-generation PDE4 inhibitor, was approved for chronic obstructive pulmonary disease by the FDA in 2011 (32). However, severe unacceptable adverse events, including nausea and vomiting, blocked the clinical application of the abovementioned PDE4 inhibitors (33). Researchers subsequently invented apremilast, a newer third-generation PDE4 inhibitor, by using a recognized functional pharmacophore of the earlier PDE4 inhibitor and adding a series of active groups in order to reduce the side effects and optimize the pharmaceutical effects (34). Apremilast has been studied on some chronic inflammatory disorders, such as psoriasis (35), dermatitis (36), Behcet’s disease (37, 38), and rheumatoid arthritis (39), appearing to have fewer side effects compared with those previous inhibitors (33). It was approved to treat adult patients with PsA and moderate-to-severe psoriasis (PsO) by the FDA in 2014 (40). Apremilast has been shown to be efficacious with an acceptable safety profile in several PsA clinical trials (40). However, no results of apremilast studies on uveitis have been reported until now (37).

In this study, using the EAU model, we first found that apremilast treatment not only significantly decreased the clinical inflammation score, including chorioretinal lesions, vascular leakage, exudate, vasculitis, and even retinal detachment of the EAU mice, but it also dwindled the degree of retinal folds and inflammatory cell infiltrations dose-dependently. These results show that apremilast could be an effective drug for AU.

The imbalance between Treg and Teff cells is the core mechanism of AU. The increase of Teff cells, especially Th17, and the decrease of Treg contributes to AU (41). In addition, the infiltration of pathological CD4+ T cells, especially Th17 and Th1, into the retina is involved in the pathogenesis of uveitis (42). Few studies have elucidated the association between PDE4 inhibitors and Treg/Th17. Zheng et al. reported that apremilast ameliorated the experimental arthritis via regulating the imbalance between Treg and Th17 cells (11).

In this study, we demonstrated that more CD4+ T-cells, especially Th17 and IFNγ-producing cells, were present in the retina of EAU mice than in the retina of the blank control mice. When apremilast was administered, the number of pathogenic cells in the retina appeared to fall. This implies that apremilast could block CD4+ T-cells, especially Teff cells, from attacking the retina in EAU. Additionally, we found that apremilast could rebuild the balance of Treg and Teff both in vivo and in vitro. Compared with the group without treatment, the apremilast group obviously upregulated the population and frequency of Treg and downregulated Th17 and Th1. For the first time, we determined that, in the EAU model, apremilast suppresses Th1 and Th17 cells and enhances Treg cells. These results were consistent with the discovery in the study by Zheng et al., who reported that apremilast ameliorated experimental arthritis via regulating the imbalance between Treg and Th17 cells (11).

PI3Ks are a family of heterodimeric lipid kinases and can be divided into three classes based on different stimuli (43, 44). AKT is one kind of serine protein kinase from the protein kinase AGC subfamily and acts as one of essential downstream factors of PI3K (44, 45). PI3K/AKT is widely regarded as an important pathway to trigger several biochemical reactions that are closely related to metabolism and disease occurrence (46). Activation of this pathway starts by different and initial upstream cell-surface receptors, including growth factor, antigen, costimulatory cytokine, chemokine, and Toll-like receptors (TLRs) (44). Once stimulated, PI3K is phosphorylated and activated, which then catalyzes the formation of the second messenger phosphatidylinositol-3,4,5-triphosphate (PIP3) (43). Then, AKT is phosphorylated to be activated by PIP3 binding together with PDK1 and AKT. Once activated, AKT phosphorylates many downstream targets, including FoxO1 (47). FoxO1 transcriptionally mediates pathways responsible for many metabolic diseases that are translated by the *FoxO1* gene, a member of *FoxO* genes belonging to the transcription factor (TF) family (48). The PI3K/AKT pathway has been reported to be important for Treg development both in vitro and in vivo. Factors downstream of AKT activation, particularly the FoxO1 transcription factors, have also been reported to play important roles in Treg development. The involvement of PI3K/AKT/FoxO1 in CD4+ T-cell differentiation by maintaining Foxp3 expression has been proven (24, 49). FoxO1 resides in the nucleus of Tregs, where it helps maintain Foxp3 expression. When phosphorylated by AKT, they are excluded from the nucleus and, thus, not able to regulate their transcriptional targets (24). PI3K/AKT are often dysregulated in autoimmune disease, and the expression of a constitutively active form of AKT leads to autoimmunity. Some autoimmune diseases, such as rheumatoid arthritis (50) and multiple sclerosis (51) show increased activity of the PI3K/AKT pathway (31). However, few studies have determined the function of the PI3K/AKT/FoxO1 pathway in the pathogenesis of uveitis. Yang et al. found that, compared with the peripheral blood mononuclear cells (PBMCs) of healthy controls, those from AAU patients with AS revealed a higher FoxO1 by a PCR-restricted fragment length polymorphism (RFLP) assay. However, further research concerning the role of FoxO1 and the biochemical pathways that control T-cell homeostasis is urgently needed to elucidate their role in the development of AAU with AS (52).

In this study, we first found that cells isolated from EAU showed higher PI3K/AKT/FoxO1 phosphorylation compared with cells from the blank control mice. In addition, apremilast prevented PI3K, AKT, and FoxO1 from phosphorylating. Furthermore, the in vitro study showed that SC79, an AKT activator, promoted the expression of inflammatory mediators, IL17A, IFNγ, and TNFα while inhibiting Foxp3, which indicated that the function of CD4+ T-cells could be regulated by the phosphorylation of intracellular AKT. Thus, we could presume that apremilast alleviates EAU by suppressing the phosphorylation of PI3K/AKT/FoxO1.

On all these counts, apremilast could alleviate EAU significantly by rebuilding the balance of Treg/Teff CD4+ T-cells via phosphorylating and activating the PI3K/AKT/FoxO1 pathway. Obviously, these results enriched our understanding of the mechanism of EAU and provided a new convincing option for the treatment of AU.

## Data Availability Statement

The original contributions presented in the study are included in the article/supplementary materials. Further inquiries can be directed to the corresponding authors.

## Ethics Statement

The animal study was reviewed and approved by the Ethics Committee of Zhongshan Ophthalmic Center, Sun Yat-Sen University.

## Author Contributions

YC was responsible for the conception and design of the study, experiments, data collection, and manuscript writing. ZL and HL provided guide of experiments. WS helped in the experiment design. YX and YP were helpful in manuscript writing. XC and DL were responsible for conception and design, revision of the manuscript, and final manuscript approval. All authors contributed to the article and approved the submitted version.

## Funding

This work was supported by grants from the National Science Foundation of China (8187040615, Guangzhou, Guangdong, China).

## Conflict of Interest

The authors declare that the research was conducted in the absence of any commercial or financial relationships that could be construed as a potential conflict of interest.
